# Expanding access to evidence-based psychotherapy in VA settings: implementation of the brief cognitive behavioral therapy for depression program

**DOI:** 10.3389/frhs.2023.1210286

**Published:** 2023-10-13

**Authors:** Joseph Mignogna, Derrecka Boykin, Raquel D. Gonzalez, Andrew Robinson, Darrell Zeno, Shubhada Sansgiry, Jennifer Broderick-Mcdaniel, Richard B. Roberson, Kristen Sorocco, Jeffrey A. Cully

**Affiliations:** ^1^ Rocky Mountain Mental Illness Research, Education, and Clinical Center (MIRECC) for Suicide Prevention, Rocky Mountain Regional VA Medical Center, Aurora, CO, United States; ^2^ Department of Physical Medicine and Rehabilitation, University of Colorado Anschutz Medical Campus, Aurora, CO, United States; ^3^ HSR&D Center for Innovations in Quality, Effectiveness, and Safety, Michael E. DeBakey VA Medical Center, Houston, TX, United States; ^4^ VA South Central Mental Illness Research, Education and Clinical Center, Michael E. DeBakey VA Medical Center Houston, TX, United States; ^5^ Menninger Department of Psychiatry and Behavioral Sciences, Baylor College of Medicine, Houston, TX, United States; ^6^ Department of Medicine, Section of Health Services Research, Baylor College of Medicine, Houston, TX, United States; ^7^ VISN 17 Primary Care Mental Health Integration, VA Heart of Texas Health Care Network, Arlington, TX, United States; ^8^ Primary Care Mental Health Integration, Audie L. Murphy VA Hospital, South Texas Veterans Health Care System, San Antonio, TX, United States; ^9^ Oklahoma Veterans Affairs Medical Center, Oklahoma City, OK, United States; ^10^ University of Oklahoma Health Sciences Center, Oklahoma City, OK, United States

**Keywords:** rural, RE-AIM, evidence-based psychotherapy, veterans, implementation science, brief CBT, telehealth, depression

## Abstract

**Introduction:**

Evidence-based psychotherapies (EBPs) are effective for mental health conditions, but access to these services remains limited and rural Veterans are particularly underserved. Specialized implementation and dissemination programs are needed to improve access to known EBPs.

**Methods:**

The current project sought to improve access to a known EBP—brief Cognitive Behavioral Therapy for depression (Brief CBT). Diverse Veterans and those from rural settings were a focus of this work. Aligned with the RE-AIM framework, a multifaceted implementation program was used to train and support VHA providers in their use of Brief CBT in VHA mental health settings, with specific outreach efforts made to providers at VHA Community-Based Outpatient Clinics (CBOCs) where rural Veterans often receive care. Evaluation included all facets of RE-AIM with a particular focus on adoption, effectiveness, and maintenance.

**Results:**

During the first two years, over 40 VHA facilities adopted the program across four regional networks. Eighty-three providers were approached, and 54 (65.1%) providers completed the training and are delivering the intervention. A total of 688 Veterans, 174 rural (25.7%), received 2,186 sessions (average of 3.5 sessions per Veteran). Veterans receiving Brief CBT with elevated depression scores who completed three or more sessions were found to have significant symptom reductions of 4.6 points (first to last available evaluations).

**Discussion:**

Implementation efforts of Brief CBT resulted in rapid uptake and significant clinical impact on Veterans. Rural outreach efforts, including targeted training for CBOC providers and use of tele-mental health, enhanced availability of EBP services for rural Veterans.

## Introduction

The Veterans Health Administration (VHA) has invested in multiple national evidence-based psychotherapy (EBP) rollouts (1–6) to improve access to high-quality mental health services. EBP trainings often involve a multi-day workshop, post-workshop supervision/consultation, and fidelity assessments and session audio reviews. Although these efforts have led to an increased number of trained providers, delivery of EBPs remains limited and rural Veterans are often negatively affected (7–10).

Factors contributing to underutilization of EBPs are often complex and occur at the patient, provider, and organizational levels (11–13). Consequently, engaging clinical and operational leaders early in the implementation and dissemination process appears to be critical to ensuring EBP uptake and sustainability (14–18). Equally important is engaging providers early and often when implementing EBPs (19) as a critical breakdown in EBP application occurs between training and its use in clinical settings (7).

This paper reports findings from a demonstration project that used a multifaceted implementation program to train and support VHA providers in delivering a brief Cognitive Behavioral Therapy for depression, known as Brief CBT (20). Funding from VHA Office of Rural Health (ORH) allowed the project to target providers at Community-Based Outpatient Clinics (CBOCs), where rural Veterans often receive care. The RE-AIM framework (21) enabled us to capture and continuously monitor the impact of this demonstration project, specifically, on the program's ability to train and support providers in the delivery of Brief CBT (adoption), Veteran depression clinical outcomes (effectiveness), as well as continued use of the program at VHA facilities over time (maintenance). Although not primary evaluation factors, additional data on reach and implementation were collected.

## Methods

### Overview

The current project sought to enhance delivery of Brief CBT using a multi-faceted provider training and support program. Brief CBT (3–6 sessions) is an empirically supported individual therapy program, designed to improve Veteran depression (22, 23). With a focus on implementation and quality improvement, this project was deemed exempt from IRB review by the Houston VA's Research and Development Office. Data for the current manuscript included training and services delivered between February 1, 2020 and December 31, 2022.

### Planned implementation sites

The Brief CBT implementation program was supported by two Veterans Integrated Service Networks (VISNs; VHA's regional organizational structure), in the South-Central United States. The first and second provider training cohorts focused on primary care mental health program providers in one VISN. Following this, a third training cohort targeted psychotherapy providers (e.g., psychologists, social workers) in primary care and CBOCs in both VISNs. A fourth cohort targeted rural and CBOC providers from either VISN. Among the planned implementation sites, there were 8 VHA Medical Centers and 29 CBOCs, for a total of 37 VHA facilities.

### Multicomponent implementation strategy

The integrated-Promoting Action on Research Implementation in Health Services (i-PARIHS) framework was used to guide our implementation strategy (24). I-PARIHS posits successful implementation as a function of four components (i.e., facilitation, innovation, recipients, and context). Facilitation was the primary mechanism driving the Brief CBT provider and support program with focused attention to help providers adopt the program within their specific clinical context.

The resulting Brief CBT provider training and facilitation program was designed for primary care and CBOC settings. The multifaceted strategy included treatment materials (i.e., provider manuals and patient workbook), web-based provider training (i.e., online and modular based training), consultation and support, as well as clinical and delivery resources including an electronic progress note template and a web-based Brief CBT delivery dashboard. These components are detailed below.

#### Treatment materials

A hardcopy and electronic links to the treatment manual and patient workbook were given to all providers. While core components of the treatment have not been altered, contextual modifications to the treatment components were made as part of the lead up to the current demonstration project to increase the likelihood of provider adoption and ease of use. For example, an abbreviated treatment manual showing a condensed view of each session's content was created to assist providers in delivering the treatment with fidelity.

#### Web-based brief CBT provider training curriculum

Training consisted of five one-hour modules and included the opportunity to earn professional educational credits. Using a standardized process for assessing and collaboratively discussing training needs with each provider, training modules were selected in collaboration between providers and Brief CBT consultants to “right size” the training experience based on provider needs. Once the curriculum was determined, providers were encouraged to complete the selected trainings within 2–3 weeks.

#### Facilitation (hereafter referred to as consultation)

Providers met with a Brief CBT consultant through individual video conference calls (15–20 min) monthly for 4–5 months. Consultation focused on reinforcing learned information from training modules, resolving implementation challenges, and providing clinical consultation. Providers were encouraged to use the electronic progress note and deliver measurement-based care using the Patient Health Questionnaire-9 (PHQ-9) at each session.

#### Clinical and delivery resources

A Brief CBT electronic progress note was created for use in the VHA's electronic health records system. The template was designed to assist providers in quickly and accurately documenting Brief CBT sessions (e.g., pre-populated radio buttons to select content delivered and measurement-based care elements—i.e., PHQ-9 and Columbia Suicide Rating Scale).

A delivery dashboard was developed to capture and share real-time data collected through the Brief CBT electronic progress note with Brief CBT providers and other relevant operational partners. The dashboard included: number of unique patients served, number of Brief CBT sessions delivered, number of sessions with a PHQ-9 administered, and depression outcome change scores as assessed by the PHQ-9.

### RE-AIM

The RE-AIM framework (21) was selected to guide evaluation across all dimensions, however, focused on adoption, effectiveness, and maintenance. Adoption was defined as the number of providers trained and using the program in clinical practice. Effectiveness was defined as within patient depression score (i.e., PHQ-9) changes during treatment. Maintenance was defined as the number of VHA facilities engaged that continued to use the program in the final six months data were captured for this paper. Secondary outcomes examined Reach (ability of the program to reach diverse Veterans, especially, those from rural settings subset of Veterans) and Implementation (degree to which the program was delivered as intended).

## Data collection methods

### Clinical and delivery data

Primary programmatic data were collected using information from the Brief CBT electronic progress note, including session type, session length, delivery modality, and specific Brief CBT content. In addition, note template data included information on PHQ-9 items and total scores as well as linking those scores to basic demographic data (e.g., age, race/ethnicity, gender), as reported in VHA electronic health records database.

### Provider feedback: survey and interview data

Survey questions, and follow-up interviews, were used to solicit feedback from providers in the earlier training cohorts. Cohort 1 (*n* = 10 providers) were contacted via email and asked if they were willing to share “what worked well and what we can improve about our program to better meet the needs of … providers.” Once scheduled, providers received a pre-survey via e-mail to complete and return prior to the interview. The survey consisted of 16 questions divided into three sections: (1) effectiveness of provider training and consultation, (2) delivery (e.g., usability of intervention materials), and (3) program impact (e.g., overall impact on patients). Questions included Likert-scale response options, rating aspects of Brief CBT from 5 (No improvements needed/Excellent) to 1 (Major improvements needed/Poor). A brief (20–40 min) semi-structured interview after the survey allowed providers to elaborate on their ratings. The interviewer took notes, and immediately after, edited for clarity. Interview notes and qualitative responses included in the survey-portion were deductively coded using the RE-AIM framework.

The second cohort of providers (*n* = 9) were asked to complete the same survey using an online portal. Given data saturation from interviews completed with the first cohort, this cohort was only provided with open-ended text boxes to provide written feedback and an option to schedule an individual interview to share verbal feedback. Mean and ranges for survey items were computed.

Across the two provider training cohorts, 16 of the 19 providers (84.2%) completed the survey, and 9 of the 10 (90.0%) providers in the first cohort also completed the individual semi-structured interview. No providers from the second cohort requested a follow-up individual interview.

## Results

### Adoption

Brief CBT adoption occurred at 41 VHA facilities (9 Medical Centers and 32 CBOCs) across four VISNs. In addition to our planned implementation sites, sites at two non-planned VISNs (i.e., one VHA Medical Center and three CBOCs) were added to the current demonstration project after their leadership expressed interest in their providers using Brief CBT. Overall, 83 providers were approached, and from these, 54 (65.1%) completed the training and planned to adopt Brief CBT into their practice. Of these, Brief CBT was successfully adopted by 44 providers working across 37 VHA facilities (i.e., eight VHA Medical Centers and 29 CBOCs within four VISNs). In contrast, 10 of the 54 providers working at seven of the planned implementation sites (i.e., four VHA Medical Centers and three CBOCs, across two VISNs) did not complete the consultation process after completing the training.

Survey items about *Adoption* assessed providers evaluations about the 1) *Brief CBT Needs Assessment and Tailored Curriculum Plan*, 2) *Web-based Training Portal*, and 3) *Consultation Sessions*. Mean scores were 4.56, 4.63, and 4.63, respectively. Similarly, an item regarding preparedness in delivering Brief CBT to patients after the consultation program ended had a mean rating of 4.56. Providers viewed Brief CBT as a good fit for their specific clinical setting (*x¯* = 4.63) and job responsibilities (*x¯* = 4.44) and regarded the treatment materials positively (*x¯* = 4.69). Provider survey open-ended responses suggested the satisfaction with training and consultation program (e.g., “I really enjoyed the program and the support the calls offered”; PC-MHI Provider #11), identified some challenges associated with completing the online training (e.g., “Sitting in front of TMS modules is pretty tough”; PC-MHI Provider #8), and provided suggestions for further adaptations (e.g., “I would suggest for the workbook to be shorter and for the templates to have more options on the check marks since some of them were repetitive and/or did not apply”; CBOC Provider #10)

Finally, providers shared information related to *Adoption* during individual interviews. Providers commented that the treatment worked well in their clinical settings. One provider stated, “This is now a part of my toolkit and I talk to patients about it on a consistent basis as part of a list of treatment options. The nice thing is that all the different options I also talk about tend to be already combined into the program in some way. So normally I would offer all the treatment options separately, but now the program incorporates most treatment options into one simple program so it's great” [PC-MHI Provider #3]*.* Other providers commented, “The flexibility was also great—certain [patients]… I’m seeing may have depressive symptoms due to chronic pain, or anxiety, or lack of sleep. I could flexibly use different parts of the treatment. Also the cards for the physical health component were great” [PC-MHI Provider #9]*.* This provider went on to comment about how their “…patients really like the workbook, it's easy to follow, the pictures, stories, and everything that's in there is great*.*” While the treatment was praised for its flexibility, one provider advised increasing its adoption potential by editing the manual “to allow for more room to adapt it or give multiple options for things so you can choose which one you like the most as the psychotherapist” [PC-MHI Provider #3].

### Effectiveness

Treatment outcomes were captured using PHQ-9 total change scores (see Table 1). These outcome data were included for patients with two or more PHQ-9 scores, among those with an initial PHQ-9 score of 10 or greater, indicating the presence of at least mild-moderate depressive symptoms. Among these patients (*n *= 263), the impact of treatment was statistically significantly different among those who completed 3 or more sessions [*χ*2(1, *N* = 263) = 5.16, *p *= .02] or 4 or more [*χ*2(1, *N* = 263) = 4.66, *p *= .03] when compared to those with fewer sessions. Additionally, while this demonstration project was not powered to test for differences, data suggest equivocal outcomes across gender (*p *= .14), race (*p *= .08), ethnicity (*p *= .30), and rurality status (*p *= .90).

**Table 1:** Treatment outcomes for patients with two or more PHQ-9 scores with an initial PHQ-9 score of 10 or greater.

Number of Sessions	PHQ-9 change score (first to last available evaluations)			
	*N* (%)	PHQ9 change score: where # sessions ≥ listed	PHQ9 change score: where # sessions < listed	*p*-value
2 or more sessions	263 (100)		–	–
Mean (SD)		4.2 (±6.1)		
Median (IQR)		−4.0 (8.0)		
3 or more sessions	211 (80.2)			0.02
Mean (SD)		−4.6 (±6.0)	−2.6 (±6.1)	
Median (IQR)		−5.0 (8.0)	−3 (6.0)	
4 or more sessions	169 (64.3)			0.03
Mean (SD)		−4.8 (±6.2)	−3.1 (±5.7)	
Median (IQR)		−5.0 (8.0)	−3.0 (7.0)	
5 or more sessions	135 (51.3)			0.09
Mean (SD)		−4.9 (±5.8)	−3.4 (±6.3)	
Median (IQR)		−5.0 (8.0)	−4.0 (8.0)	
Demographic Comparisons (*n* = 263)		PHQ9 change (Mean (SD))	PHQ9 change (Median (IQR))	*p*-value
Gender				0.14
Male	218 (82.9)	−3.9 (6.3)	−4.0 (8.0)	
Female	45 (17.1)	−5.4 (4.6)	−6.0 (5.0)	
Race				0.08
White	160 (60.8)	−3.6 (6.2)	−4.0 (7.5)	
African American	71 (27.0)	−4.5 (5.9)	−4.0 (9.0)	
Others	32 (12.2)	−6.4 (5.3)	−5.5 (5.5)	
Ethnicity				0.30
Hispanic or Latina/x	89 (33.8)	−4.6 (7.1)	−6 (10.0)	
Non-Hispanic or Latina/x	113 (43.0)	−3.9 (5.2)	−4.0 (6.0)	
Unknown	61 (22.2)	−4.1 (5.9)	−5.0 (7.0)	
Rurality Status (*N*, %)				0.90
Rural	75 (28.5)	−4.5 (5.6)	−5.0 (7.0)	
Non-Rural/Urban	184 (70.0)	−4.1 (6.3)	−4.0 (8.0)	
Unknown	4 (1.5)	−2.8 (7.4)	−3.5 (9.5)	

Providers' ratings on survey items related to *Effectiveness* were positive. Specifically, the statement, *Overall impact on patients* had a mean rating of 4.25. The statement, *Impact on patient depression scores* had a mean rating of 4.13. One provider wrote, “I love these interventions and how easy and meaningful they are” [CBOC Provider #10]. Another provider commented about how the PHQ-9 did not capture all patient improvements on depressive symptoms, stating, “Sometimes people report functional improvement, but their PHQ-9 scores may stay the same. When discussing this with the patient, I don't really get any more clarity on why that is the case” [PC-MHI Provider #1].

During individual interviews, providers described how Brief CBT had a positive impact on patients during and after Brief CBT. One provider stated: “This training has improved my overall effectiveness with patients. In a couple of sessions, with some patients, they were good to go. For example, one patient realized after a couple sessions, ‘My feelings are not always representative of reality—now I see where I was making a mistake. I was letting my emotions lead,’ and that was enough for her” [PC-MHI Provider #3].

### Maintenance

Of the planned implementation sites (37 facilities across 2 VISNS), all facilities (100%) continue to have one or more providers delivering Brief CBT in the final six months data were captured for this paper. Survey items related to *Maintenance* included provider responses to the question, *Impact on your own professional development*. This item had a mean rating of 4.44. In interviews, providers expressed their desire to continue to use Brief CBT over time. One provider wrote, “Once a tool, always a tool. I would never let go of anything like this [PC-MHI Provider #6], and another stated “Still using this program and still planning on using it and moving forward with it. I always keep checking with my patients to make sure they enjoy it” [PC-MHI Provider #7]. One provider expressed a desire to see Brief CBT implemented more broadly across the VHA, stating, “I believe it should be rolled out to other [VHAs], very useful. Would be great to roll out to other [VHAs] and other VISNS in terms of saving time for other providers. Sometimes we have 10–12 patients per day. Things like this program are key and are useful to make sure the patient is on board for treatment and benefitting from the treatment” [PC-MHI Provider #9]*.* Another provider described the expected impact of using Brief CBT going forward, saying, “I think it's great and definitely something that would be more helpful for providers by giving structure to treatment and having helpful things delivered every session. That way it cuts down on only supportive therapy” [PC-MHI Provider #1].

### Reach

Patients receiving brief CBT were racially and ethnically diverse (see Table 2). Treatment was delivered to 688 unique patients (25.3% rural), with an average age of 49.5 (SD = 16.5) years, and consistent with the VHA patient population, mostly males (79.4%). While a majority were White (65.0%) and Non-Hispanic or Latina/x (41.6%), racial and ethnic diversity were represented, with 21.2% identified as African American and 33.6% as Hispanic or Latina/x.

**Table 2:** Patient demographics.

Descriptive Data	*N* (%)
Number of Unique Patients	688 (100)
Age	
Mean (SD)	49.5 (±16.5)
Median (IQR)	49.0 (IQR 28.0)
Gender	
Male	546 (79.4)
Female	142 (20.6)
Race	
White	447 (65.0)
African American	146 (21.2)
Others and Unknown	95 (13.8)
Ethnicity	
Hispanic or Latina/x	231 (33.6)
Non-Hispanic or Latina/x	286 (41.6)
Unknown	171 (24.9)
Rurality status (*N*, %)	
Rural	174 (25.3)
Non-rural/urban	502 (73.0)
Unknown	12 (1.7)
Delivery Data	visits = 2,186 (100%)
Total number of sessions	
Clinic Stop	
Number (%) of face-to-face sessions	822 (37.6)
Number (%) of telephone sessions	215 (9.8)
Number (%) of video sessions	1,149 (52.6)

Demographic and clinical assessment data were captured by accessing the VHA's electronic health records database. Categories reported here are consistent with how they are coded in this database. Demographic data are initially captured when a Veteran first registers for VHA care, and subsequently updated as needed.

Modality of Brief CBT delivery, a potentially critical factor in enhancing intervention reach, was also diverse, with over half of sessions delivered by video telehealth (52.6%). Other delivery modalities included face-to-face (37.6%) and telephone (9.8%). Also of note, some providers adapted Brief CBT for delivery in group format, represented by 4.4% of the total sessions delivered (see Table 3).

**Table 3:** Brief CBT delivery data.

Length of Session, *N *= 2,186 (100%)	
30 min (*N*, %)	1,387 (63.5)
45 min	342 (15.7)
60 min	228 (10.4)
Group	97 (4.4)
Other	132 (6.0)
Sessions per Patient	
Avg. number of bCBT sessions per Patient	
Mean (SD)	3.5 (±2.5)
Median (IQR)	3 (IQR 4)
Number of sessions, *N *= 688 (%)	
1–2 sessions	314 (45.6)
3–4 sessions	152 (22.1)
≥5 sessions	222 (32.3)
Frequency data on number of sessions (Number (%) of Patients)	
1 session	200 (29.1)
2 sessions	114 (16.6)
3 sessions	85 (12.4)
4 sessions	67 (9.7)
5 sessions	68 (9.9)
6 or more sessions	154 (22.4)

From the provider survey, items evaluating *Reach* assessed the usability of the intervention materials for patients, responses had a mean rating of 4.5 (rating between *Good* to *No improvements needed/Excellent* descriptive anchors). The broader applicability of Brief CBT was captured in provider feedback during interviews. One provider stated, “This is not intensive so it's great for [patients]… that are ambivalent towards therapy. Like dipping your toes in the water so I think every [patient]… could use this” [PC-MHI Provider #8]. Another provider wrote, “I’ve gotten feedback from patients that it's easy to follow. It's one of those treatments that they have actually shared with family or loved ones (do behavioral activation with them or getting them more involved)—gets patients excited which makes it useful. This is a very user-friendly program” [PC-MHI Provider #9]*.* Another provider commented about the benefits of the patient workbook, stating, “Patients won't read [a] very dense manual. Less words, lots of pictures, easy for them to open up and get the idea that way. The patient manual [i.e., workbook] was good like that” [PC-MHI Provider #3].

### Implementation

Implementation outcomes focused on if treatment was delivered as intended, and what, if any, modifications were required to implement it into practice. While fidelity to the treatment protocol was not directly assessed, other delivery data were used as measures of fidelity. First, the target length of each session was designed to be delivered in as little as 30 min to accommodate providers in primary care settings. Consistent with this, delivery data indicate 63.5% of sessions were delivered in this amount of time (see Table 3). Additionally, the target number of Brief CBT sessions, as determined by prior randomized trials (22), was three or four. In the current sample, the average number of sessions across all patients was 3.5 (*SD* = 2.5) with 22.1% (*n *= 152) of all patients receiving exactly 3 or 4 sessions.

Provider use of measurement-based care practices was viewed as an additional construct of intervention fidelity. Delivery data indicate 85.5% (*n* = 588) of patients with Brief CBT sessions had at least one PHQ-9 collected. Of these patients, 68.0% (*n *= 400) had two or more PHQ-9 collected. Treatment fidelity can also be assessed in relation to the care provided to urban (*x¯* = 3.6 (*SD *= 2.4) sessions) vs. rural (*x¯* = 3.5 (*SD *= 2.6) sessions) patients (see Table 4). Chi-square test comparing clinically meaningful groupings of number of treatment sessions (i.e., 1–2, 3–4, or 5 or more sessions) for rural vs. urban patient groups was not significant (*χ*2(2, *N* = 676) = .057, *p* = .972); suggesting that rural Veterans received a similar amount of care relative to their urban counterparts.

**Table 4:** Comparison of rural and urban patients.

Comparison of number of sessions (*n *= 676)^a^	Rural *n* (%)	Urban *n* (%)
	*N *= 174 (25.7)	*N *= 502 (74.3)
Avg. number of Brief CBT sessions per patient (mean, *SD*)	3.6 (2.4)	3.5 (2.6)
Total Sessions Received		
1–2 sessions	77 (44.3)	227 (45.2)
3–4 sessions	39 (22.4)	112 (22.3)
≥5 sessions	58 (33.3)	163 (32.5)
Frequency of sessions		
1 session	43 (24.7)	150 (29.9)
2 sessions	34 (19.5)	77 (15.3)
3 sessions	23 (13.2)	62 (12.4)
4 sessions	16 (9.2)	50 (10.0)
5 sessions	19 (10.9)	49 (10.0)
6 or more sessions	39 (22.5)	114 (22.4)
Note: Numbers below are not mutually exclusive. One patient can have different clinic stops and sessions type over time (visits = 2,186)	Rural *n* (%)	Urban *n* (%)
	*N *= 553 (25.3)	*N *= 1,610 (73.7)
Total number of sessions		
Number of face-to-face sessions	215 (38.9)	597 (37.1)
Number of telephone sessions	79 (14.3)	132 (8.2)
Number of video sessions	259 (46.8)	881 (54.7)
Length of Session		
30 min (*n*, %)	279 (50.5)	1,086 (67.5)
45 min	123 (22.2)	219 (13.6)
60 min	66 (11.9)	161 (10.0)
Other	67 (12.1)	65 (4.0)
Group	18 (3.3)	79 (4.9)

^a^ Data for 12 participants were not included in this table as these data were missing from their electronic health records.

Providers had generally positive ratings on the survey related to *Implementation*. Specifically, the item that assessed the usability of the CPRS note template had a mean rating of 4.31. Written comments from one provider describe a preference for the “workbook to be shorter” and shared ideas for making the electronic progress note template more efficient. Provider interviews suggested providers viewed Brief CBT as a “good fit*”* for PCMHI clinics. However, some providers reported experiencing some challenges around delivering treatment in a 30-min session. One provider described this challenge, particularly in relation to Session One, and the resulting impact, stating, “We only have 25–30 min to do each session, so the Session One has a lot of information to cover in there and trying to get all of that in is a little bit difficult, and if I can fit it all in I do, if I can't then I can't” [PC-MHI Provider #3]. Another provider highlighted challenges associated with delivering EBPs, stating how it was “…hard to keep the patient in a more active role—sometimes it can be difficult since we want treatment to be Veteran-centered, but also follow the protocol. If the patient comes in talking about anything and everything else, it can add some pressure” [PC-MHI Provider #7].

Appreciation was expressed for consultants’ responsiveness to feedback, noting how that made it easier to use Brief CBT in practice. One provider stated, “I would give feedback and have things updated shortly after by the team. For example, within the note template, suicide risk assessments are part of every session. The note template was updated so I could add that in easily. Consultants are responsive to what the providers need” [PC-MHI Provider #3]*.* Appreciation was also shared for how the program honored clinicians, with one provider stating, in contrast to other more structured EBP training programs, “…this program is more about ‘you are a professional, here's a guideline for the treatment and use it as you see fit’” [PC-MHI Provider #3].

## Discussion

Implementation of Brief CBT resulted in rapid uptake and significant clinical impact on patients across a large healthcare system. The Brief CBT implementation program was quickly adopted (within two years) by over 40 VHA facilities spread across 4 regional networks and sustained over time. There was substantial interest and support for the program, as 54 providers were trained and delivered over 2,186 Brief CBT sessions to a diverse group of 688 patients (average of 3.5 sessions per patient). Importantly, patient depression scores indicate significant symptom reductions of 4.6 points (first to last available evaluations) on the PHQ-9 among those receiving three or more Brief CBT sessions.

To reach rural patients, the Brief CBT Resource Team targeted training for VHA CBOCs. Additionally, tele-mental health procedures were employed to enhance the availability of EBP for rural patients. Multiple options for how to receive care might be beneficial and/or needed for rural veterans to connect. Consequently, providers were encouraged to use whatever delivery modality was most convenient for their patients. Selection appears to be, at least in part, dependent upon patient preference/needs, as treatment was most commonly engaged via video sessions.

RE-AIM provided a useful evaluative framework for ongoing assessment of implementation efforts. Solicited feedback from stakeholders (e.g., advisory council) leading up to implementation, and providers during implementation, allowed the Brief CBT Resource Team to be responsive to identified needs and challenges and success in the *Implementation* and *Reach* domains. Adaptations were made to peripheral aspects of the treatment and how it was delivered, including the following:

• Treatment materials were made more accessible to support virtual delivery (e.g., patient workbook was modified to be a fillable PDF, allowing patients to mark on these forms without requiring that they be printed), and posted on a public facing website.
• A new patient vignette was added to the patient workbook to broaden the applicability of the workbook examples for a diverse patient population.
• A “Session 0” was added to the treatment manual to accommodate the initial intake session to increase the fit of Brief CBT within the context of PC-MHI.
• Columbia Suicide Risk Assessment measure was added to the electronic progress note template to comply with VA policy around suicide risk assessment.

A significant challenge to multisite implementation in a large healthcare system is accounting for expected turnover of providers, for example, when newly trained providers move to different clinics or accept positions at new locations. However, this also provides a potential opportunity to spread an EBP to new sites. In the current project, two recently trained providers accepted new positions at different VHA facilities; however, they continued to use Brief CBT and/or provided consultation/supervision of colleagues/trainees to deliver Brief CBT. The Resource Team reached out to these providers to offer assistance to support their efforts. Also of note, it is important to work with local and regional leadership to support an infrastructure capable of training new providers, as well as monitoring those already trained to encourage their continued use of the EBP.

We were pleased to see how Brief CBT spread by “word of mouth.” This provides another measure of *Reach* and *Adoption*. Resource Teams should develop flexible plans for how they will support unplanned implementation sites. Going forward, we are utilizing the World Health Organization ExpandNet Scaling Up Framework to guide our implementation efforts at planned and unplanned sites/regional networks (24).

Limitations should be noted. This was a clinical demonstration project, and consequently, effectiveness outcomes should be considered in this context (no control group). This project also did not allow for testing of implementation facets—so it is not clear which elements were more or less impactful on delivery and adoption.

## Conclusion

This paper reported findings of a demonstration project using a multifaceted implementation program to train and support providers to deliver a brief psychotherapy for depression, that included focused efforts to support providers serving rural Veterans. RE-AIM provided a useful evaluative framework that allowed for ongoing assessment of implementation outcomes, particularly outcomes related to adoption, effectiveness, and maintenance. Program outcomes suggest that targeted implementation efforts to train and support VHA CBOC providers generated high rates of adoption, clear and positive impact on rural Veteran outcomes, and suggest that the program is sustainable over time.

## Data Availability

The datasets presented in this article are not readily available because patient electronic health records data are owned by the Veterans Health Administration and the corresponding author does not have the authority to share these raw anonymized data. The interview and survey data generated by providers are not publicly available because these data contain potentially identifying information, and providers were assured that the information they provided would be publicly available only in aggregate. Requests to access the datasets should be directed to Office of Research and Development, Department of Veterans Affairs.
